# Genetic Kidney Diseases (GKDs) Modeling Using Genome Editing Technologies

**DOI:** 10.3390/cells11091571

**Published:** 2022-05-06

**Authors:** Fernando Gómez-García, Raquel Martínez-Pulleiro, Noa Carrera, Catarina Allegue, Miguel A. Garcia-Gonzalez

**Affiliations:** 1 Laboratorio de Nefroloxía (No. 11), Grupo de Xenética e Bioloxía do Desenvolvemento das Enfermidades Renais, Instituto de Investigación Sanitaria de Santiago (IDIS), Complexo Hospitalario de Santiago de Compostela (CHUS), 15706 Santiago de Compostela, Spain; fernando.gomez.garcia@sergas.es (F.G.-G.); raquel.martinez.pulleiro0@usc.es (R.M.-P.); noa.carrera.cachaza@sergas.es (N.C.); 2 Grupo de Medicina Xenómica, Centro Singular de Investigación en Medicina Molecular y Enfermedades Crónicas (CiMUS), 15706 Santiago de Compostela, Spain; 3 Fundación Pública Galega de Medicina Xenómica-SERGAS, Complexo Hospitalario de Santiago de Compostela (CHUS), 15706 Santiago de Compostela, Spain

**Keywords:** Genetic kidney diseases, ZFN, TALEN, CRISPR-Cas9, disease modeling, GKDs models

## Abstract

Genetic kidney diseases (GKDs) are a group of rare diseases, affecting approximately about 60 to 80 per 100,000 individuals, for which there is currently no treatment that can cure them (in many cases). GKDs usually leads to early-onset chronic kidney disease, which results in patients having to undergo dialysis or kidney transplant. Here, we briefly describe genetic causes and phenotypic effects of six GKDs representative of different ranges of prevalence and renal involvement (ciliopathy, glomerulopathy, and tubulopathy). One of the shared characteristics of GKDs is that most of them are monogenic. This characteristic makes it possible to use site-specific nuclease systems to edit the genes that cause GKDs and generate in vitro and in vivo models that reflect the genetic abnormalities of GKDs. We describe and compare these site-specific nuclease systems (zinc finger nucleases (ZFNs), transcription activator-like effect nucleases (TALENs) and regularly clustered short palindromic repeat-associated protein (CRISPR-Cas9)) and review how these systems have allowed the generation of cellular and animal GKDs models and how they have contributed to shed light on many still unknown fields in GKDs. We also indicate the main obstacles limiting the application of these systems in a more efficient way. The information provided here will be useful to gain an accurate understanding of the technological advances in the field of genome editing for GKDs, as well as to serve as a guide for the selection of both the genome editing tool and the gene delivery method most suitable for the successful development of GKDs models.

## 1. Introduction

Rare kidney diseases comprise more than 150 different diseases, with a global prevalence of around 60 to 80 cases per 100,000 individuals, and most of them have a genetic etiology (Genetic kidney diseases) [1,2]. Genetic kidney diseases (GKDs) are one of the leading causes of early onset chronic kidney disease (CKD), that is, CKD manifesting before 25 years of age [3]. CKD involves reduced glomerular filtration rate and/or increased urinary albumin excretion. Complications of CKD include increased all-cause mortality, acute kidney injury and kidney-disease progression [4,5]. Furthermore, GKDs are the fifth most common cause of end-stage renal disease (ESRD) after diabetes, hypertension, glomerulonephritis, and pyelonephritis and accounts for at least 10–15% of adult kidney replacement therapy (KRT) cases [1,4]. However, thanks to the progress in KRT, GKDs patients rarely die when their disease progresses and can live for many years. Nevertheless, these patients often have compromised health with a poor quality of life [1].

**Figure 1:**
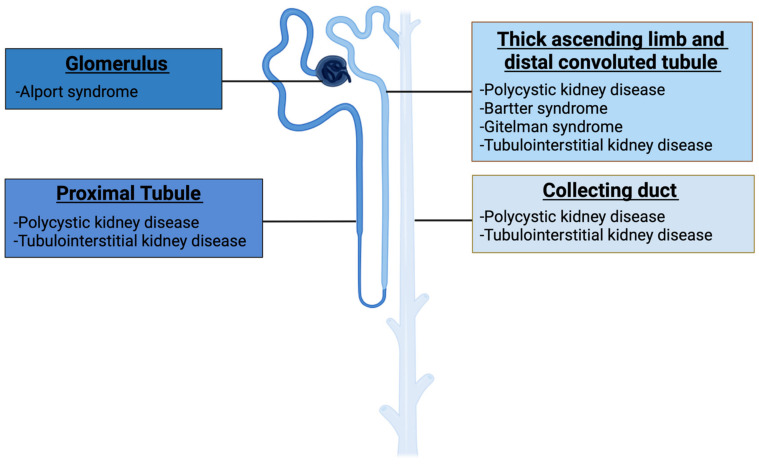
Representation of the nephron and selection of Genetic kidney diseases. Diseases are arranged and categorized according to the predominant localization and manifestation of the defect within the nephron.

Genomic analyzes to detect pathogenic variants that cause GKDs are extremely important. The development and implementation of genomic analysis techniques over the last two decades, including high-throughput next-generation sequencing (NGS), have led to the discovery of many GKDs-responsible genes. Currently, more than 600 genes are associated with GKDs [6,7]. The proteins encoded by these genes are expressed in different segments of the nephron: glomerulus, proximal tubule (PT), thick ascending limb (TAL), distal convoluted tubule (DCT), and collecting duct (CD) (Figure 1) [8].

In most cases, the presence of a mutation in one of these genes is sufficient to generate the disease, that is, most GKDs are monogenic (also termed “Mendelian”), with a strong genotype-phenotype correlation, a clear pattern of inheritance and where environmental factors have limited influence [9]. The opposite situation happens with complex or polygenic diseases, where multiple genetic variants (mostly common risk variants) at different locus and environmental factors contribute to the pathology, with a lack of simple patterns of inheritance. Common genetic variants have been shown to contribute to several kidney diseases, such as IgA nephropathy, membranous nephropathy, or nephrotic syndrome [9].

Since most GKDs are monogenic, site-specific nuclease systems could be used to edit the genes that cause these diseases. These systems include zinc finger nucleases (ZFNs), transcription activator-like effect nucleases (TALENs) and regularly clustered short palindromic repeat-associated protein (CRISPR-Cas9) which can generate nucleotide changes in the gene of interest. Therefore, using these genome editing technologies, it would be possible to modify the genes causing GKDs to study the phenotypic effects associated with such modifications [8]. This would be very useful, since there is currently a profound misunderstanding of the pathophysiology of these diseases. In addition, we will be able to answer questions about the impact of individual proteins on the development of kidney disease, the pathophysiological mechanisms that mark disease progression and possible therapeutic interventions [8].

In this review, we recapitulate and describe the different genome editing systems generally used in monogenic kidney diseases with different nephron segment deficiencies and various prevalence rates (high prevalence: autosomal dominant polycystic kidney disease and Alport syndrome; intermediate prevalence: autosomal recessive polycystic kidney disease and autosomal dominant tubulointerstitial kidney disease; and finally, low prevalence: Gitelman and Bartter syndromes). This will allow us to have updated information on actual and near future strategies for an exciting new era on GKDs research and treatment.

### 1.1. Genetic Kidney Diseases

As mentioned above, there are more than 600 genes linked to GKDs. These genes encode a wide variety of proteins including receptors, channels, enzymes, transcription factors, and structural components (Table 1) [10]. When the function of any of these proteins is compromised, vital processes such as water and electrolyte balance, blood pressure regulation, acid-base homeostasis, tissue oxygen supply, hormone and vitamin metabolism, innate and adaptive immunity, and central nervous and cognitive functions may be affected, resulting in various diseases [10].

#### 1.1.1. Autosomal Dominant Polycystic Kidney Disease (ADPKD) 

ADPKD is a monogenic disease caused mainly by mutations in *PKD1* gene (accounts approximately for 85% of cases) which encodes polycystin-1 (PC1) and *PKD2* gene (accounts approximately for 15% of cases) which encodes polcystin-2 (PC2) [12,13]. However, it can also be caused, in a minority percentage, by mutations in other genes such as *GANAβ*, *DNAJB11*, and *IFT140* [14,15,16]. PC1 and PC2 proteins are involved in the differentiation and maintenance of renal tubular cells, and are predominantly, but not exclusively, localized in the primary cilium of these cells. Therefore, this kidney disease is classified as a ciliopathy [17,18,19]. ADPKD is the most common genetic kidney disease and affects ~1/400–1/1000 individuals [20]. It is the only GKD that is not a rare disease [1,2]. In approximately 90% of patients, the condition is inherited with a positive family history, while in 10% the disorder appears to be due to a de novo mutation [21].

The kidneys of patients with ADPKD are characterized by the formation of cysts that gradually grow in number and size. In early stage of the disease, the kidney contains few fluid-filled cysts and a large amount of well-preserved parenchyma [22]. In advanced ADPKD, marked kidney enlargement, vascular remodeling, interstitial fibrosis, and hepatic cysts are observed [23]. These pathological processes contribute to the development of ESRD, which is a severe renal disease requiring dialysis or kidney transplantation for patient survival. Particularly, 50% of ADPKD patients will reach ESRD before the age of 60 years, which puts ADPKD as a major cause of ESRD [24,25].

#### 1.1.2. Autosomal Recessive Polycystic Kidney Disease (ARPKD)

ARPKD is a monogenic disease caused mainly by mutations in *PKHD1* gene which encodes polyductin (also known as fibrocystin) [26,27]. However, it can also be caused, in a minority percentage, by mutations in *DZIP1L* gene [28]. Polyductin is involved in the terminal differentiation of the CD and biliary system and is localized in the primary cilium of renal epithelial cells, so this renal disease is also classified as a ciliopathy [17,29]. Even though ARPKD is less common than ADPKD (occurs in approximately 1 in 20,000 live births) [24], it is one of the common hereditary renal cystic diseases in infants [30].

The kidneys of patients with ARPKD are characterized by the presence of innumerable minute “microcysts” (1–2 mm) along the length of the DC, involving both the cortex and medulla and resulting in marked renal enlargement. Clinical features of ARPKD include ectasia of the DCs and hepatic bile ducts with associated renal and hepatic fibrosis [31]. Approximately 50% of patients with ARPKD present with this disease as neonates [32] and are born with very large kidneys [31]. These neonates suffer a 30% mortality rate due to respiratory and/or renal dysfunction [33]. For children who survive the perinatal period, they rarely survive beyond the age of 60 years [34].

#### 1.1.3. Alport Syndrome (AS)

AS, also named type IV collagen nephropathy, is a monogenic disease caused by mutations in *COL4A3*, *COL4A4*, or *COL4A5* genes which encode collagen chains α3, α4, and α5, respectively. These collagen chains are located, among others, in the glomerular basement membrane (GBM) [35], so this kidney disease is classified as a glomerulopathy [36]. AS is the second most common genetic kidney disease, after ADPKD [6]. Although its exact prevalence is unknown, a recent population-based study on the gnomAD cohort reports a frequency of 1/2320 individuals with a predicted pathogenic mutation in *COL4A5*, 1/106 individuals with a heterozygous predicted pathogenic mutation in *COL4A3* or *COL4A4*, and 1/88,866 individuals with two heterozygous predicted pathogenic mutations in trans [37,38].

Individuals with pathogenic mutations in any of these genes can present with a wide phenotypic variability, ranging from isolated hematuria to renal failure, depending, among others, on the affected gene and if only one or the two copies of the gene are altered [39]. X-linked disease accounts for 70–75% of Alport patients [40,41,42]. Males with this condition are severely affected, with 90% probability of starting ESRD by the age of 40 years old, in addition to presenting with other extrarenal manifestations (e.g., hearing loss or ocular abnormalities) [43]. The phenotype of female carriers of heterozygous mutations in *COL4A5* is highly variable, ranging from mild phenotypes with intermittent hematuria to severe cases [44]. Pathogenic mutations in the *COL4A3* or *COL4A4* genes cause the autosomal-recessive (AR) Alport syndrome, a similar phenotype to that of X-linked inheritance in males [45,46] or the “autosomal dominant” (AD) Alport syndrome, a less severe phenotype with persistent hematuria but without extrarenal manifestations. However, a portion of them may progress to ESRD [40,45,47,48].

#### 1.1.4. Autosomal Dominant Tubulointerstitial Kidney Disease (ADTKD)

ADTKD is a monogenic disease caused by mutations in five different genes, including *UMOD*, *MUC1*, *REN*, H*NF1β*, and *SEC61A1* which encode uromodulin, transmembrane epithelial mucin 1, renin, hepatocyte nuclear factor 1β and α1 subunit of the SEC61, respectively [49]. These proteins carry out renal and extrarenal functions and their malfunctioning generates tubular damage and interstitial fibrosis, so this kidney disease is classified as a tubulopathy [49,50]. Although the exact prevalence has not been determined, data suggest that ADTKD is one of the most common monogenic kidney diseases after ADPKD and AD, accounting for ~5% of monogenic disorders causing CKD [49,51].

ADTKD is characterized by a lack of specific clinical, biological, and pathological features [50]. For instance, affected individuals present with progressive CKD, normal or slightly elevated blood pressure, and normal or small kidney size [52]. In addition, the symptoms and organs affected may vary depending on the altered gene. For example, mutations in *UMOD*, *MUC1*, and *REN* mainly affect the kidney [53], whereas mutations in *HNF1β* and *SEC61A1* also cause variable extrarenal manifestations [54,55].

#### 1.1.5. Gitelman (GS) and Bartter (BS) Syndromes

GS and BS are monogenic diseases caused by mutations in different genes [56]. While GS is an autosomal-recessive disease produced by mutations in a single gene (*SLC12A3*) [57], BS can be caused by mutations in six distinct genes (*SLC12A1*, *KCNJ1*, *CLCNKA*, *CLCNKB*, *BSND*, and *MAGED2*) and be transmitted in three inheritance patterns (X-linked, autosomal-recessive, or autosomal-dominant) [58,59,60,61,62,63,64]. The proteins encode by these genes reside in different parts along the nephron, particularly in the DCT (associated with GS) and in the TAL (associated with BS), so these kidney diseases are classified as tubulopathies [56,65]. For GS, the estimated prevalence varies from 1 to 10 per 40,000 individuals [66], whereas for BS, the data are not well-defined (but an incidence of 1 per 1,000,000 is estimated) [67].

Although GS and BS have different etiologies, they share most of their clinical symptoms. Patients with GS or BS can present different symptoms as hypokalemia, hypochloremia, metabolic alkalosis, hyperreninemia, hyperaldosteronism or even CKD [65]. However, the main difference between the two is that patients with GS also present hypocalciuria. This is due to an increase in calcium (Ca^2+^) reabsorption to compensate for the loss of salts. This compensatory process does not occur in patients with BS [68].

### 1.2. Site-Specific Nuclease Systems

Genome editing technologies have provided a sturdy tool for biomedical research with extraordinary versatility and feasibility. This technology can produce genome modifications, such as targeted mutagenesis or site-directed insertion/deletion/substitution at specific sites in the genome, using engineered nucleases. Genome editing relies on the production of site-specific double-strand DNA breaks (DSBs) and subsequent endogenous cellular repair through the error-prone nonhomologous end-joining (NHEJ) or the error-free homology-directed repair (HDR) pathway [69].

On the one hand, in NHEJ several enzymes are used to directly join the break ends of the DSBs without the need for a homologous repair template and can be carried out during any phase of the cell cycle in higher eukaryotes [70,71]. Due to its error-prone nature, NHEJ repair often leads to the insertion or deletion (InDels) of nucleotides and, thus, DNA sequence alterations at the targeted DSBs sites [69]. So NHEJ can be exploited to introduce frameshifts into the coding sequence of a gene (knock out) by a combination of two mechanisms: pre-mature truncation of the protein and nonsense-mediated decay of the mRNA transcript [71]. On the other hand, in HDR a homologous sequence serves as a template to repair the DSBs allowing an accurate repair and it occurs mainly during late S phase or G2 phase of the cell cycle [70,71]. Owing to its error-free nature, HDR can be utilized to insert or correct a specific mutation at the target loci (knock in) in the presence of an exogenously supplied donor oligo as a repair template by homologous recombination [69,71].

**Figure 2:**
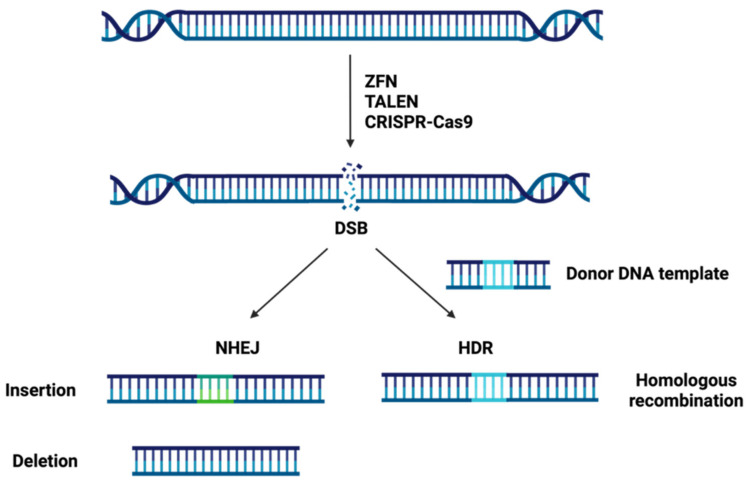
Cellular repair mechanisms of double-strand DNA breaks (DSBs). Site-specific nuclease systems carry out precise and efficient genome modification by producing a DSB, which would be corrected by nonhomologous end-joining (NHEJ) or homology-directed repair (HDR) mechanisms.

By taking advantage of the intrinsic DNA repair machinery of cellular organisms, tools that produce DSBs can be used to genome editing. The three major site-specific nuclease systems are: zinc finger nucleases (ZFNs), transcription activator-like effector nucleases (TALENs) and clustered regularly interspaced short palindromic repeats-CRISPR associated protein-9 (CRISPR-Cas9) (Figure 2).

#### 1.2.1. Zinc Finger Nucleases (ZFNs)

**Figure 3:**
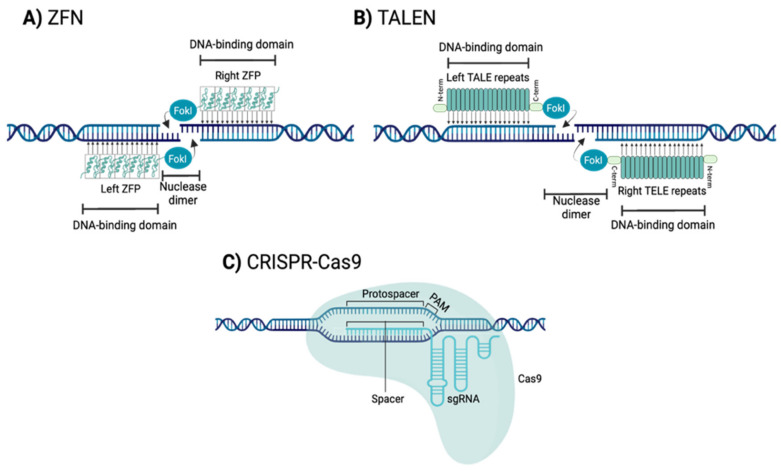
Site-specific nuclease systems. (**A**) Zinc-finger nucleases (ZFNs) recognize DNA target sequence using specific tandems of three base pair recognition motifs (ZFP). Paired ZFNs bind to the opposite strands to dimerize FokI, producing a DSB at the desired site; (**B**) transcription activator-like effector nucleases (TALENs) recognize the specific DNA sequence through TALE repeats that include repeat-variable di-residues (RVDs). TALE proteins contain an N-terminal region, a central domain of repeats and a C-terminal region. Paired TALENs bind to the opposite strands to dimerize FokI, producing a DSB at the desired site; (**C**) CRISPR-Cas9 recognizes the specific DNA sequence using a single guide RNA (sgRNA) and the Cas9 protein recognizes the protospacer adjacent motif (PAM) and cleaves the DNA at the target site, generating a DSB.

ZFNs are engineered nucleases composed of sequence-specific zinc finger DNA-binding domains and a non-specific DNA cleavage domain derived from the type II restriction endonuclease FokI (Figure 3A) [72]. The DNA-binding domain contains several linked zinc fingers (ZF) motifs, and each motif consists of approximately 30 amino acids in a conserved ββα secondary configuration [73]. Each individual ZF motif recognizes a 3-base pair (bp) specific DNA sequence and ZF motifs have been developed to recognize all of the 64 (4 x 4 x 4) possible nucleotide triplets [69,74]. This approach allows a specific tandem ZF motifs (ZFP) to potentially bind to a nucleotide sequence (typically with a length that is a multiple of 3, usually 9 bp to 18 bp) that is unique within a cell’s genome [71].

To induce a cleavage at a single and specific site in the genome two individuals ZFPs are required [75]. ZFPs must be designed as a pair able to recognize two sequences flanking the target site, one on the forward strand and the other on the reverse strand. Upon binding of the ZFPs, the pair of FokI domains dimerize and cleave the DNA at the target site, generating a DSB [76].

#### 1.2.2. Transcription Activator-Like Effector Nucleases (TALENs)

TALENs are engineered nucleases composed of a transcription activator-like effector (TALE) DNA binding protein and a non-specific DNA endonuclease FokI (Figure 3B) [77]. TALE protein contains an N-terminal translocation signal, a C-terminal acidic transcription-activation domain, and a central DNA-binding domain [78]. The DNA-binding domain is composed of TALE repeats. Each repeat is 33 to 35 amino acids in length, including two relevant amino acids (termed the repeat-variable di-residue (RVD)) conferring specificity for one of the four DNA base pairs [78]. Therefore, each of the TALE repeats binds to a single nucleotide [69].

This direct relationship between TALE repeat and nucleotide recognition allows a specific tandem of TALE repeats to potentially bind to a single nucleotide sequence in a cell’s genome (typically 18-bp or even more) (typically 18 bp or even more) [69,75,79]. Like ZFNs, to induce a cleavage at a single and specific site in the genome two TALENs monomers are required to recognize flanking sequences of the target site. Upon binding of the TALENs on either side of the target site, the pair of FokI domains dimerize and cleave the DNA at the target site, generating a DSB [69,71].

#### 1.2.3. Clustered Regularly Interspaced Short Palindromic Repeats-CRISPR Associated Protein 9 Nuclease (CRISPR-Cas9)

CRISPR-Cas9 are engineered systems composed of a Cas9 endonuclease, which is used to cleave the target sequence, and a single guide RNA (sgRNA), which defines the specificity of the Cas9 and guides Cas9 to the target DNA (Figure 3C) [80,81]. The sgRNA presents a sequence of 20 bp (spacer) nucleotides that are complementary to the target genomic sequence (protospacer) and a scaffold region that allows its association with Cas9. This association produces a conformational change in Cas9, which goes from a self-inhibited state to a functionally active state [82,83,84].

Activated Cas9 recognizes, in the target genome, a sequence of three nucleotides called protospacer adjacent motif (PAM). This PAM is different for each type of Cas9 (e.g., for *Streptococcus pyogenes*-SpCas9 it is (N)GG, where N is any nucleotide) [83,84]. Once the PAM sequence is recognized, the double strand dissociates. If the sgRNA spacer sequence is complementary to the target nucleotides adjacent to the PAM (protospacer), then the Cas9 cleaves the DNA at the target site, generating a DSB [82].

It should be noted that the CRISPR systems are continuously improving. New Cas9s have been discovered in other bacteria, such as *Staphylococcus aureus*-SaCas9 and *Francisella novicida*-FnCas9. In addition, new SpCas9s have been developed that offer improved efficiency and specificity, as well as being able to recognize different PAMs (e.g., SpCas9-NRRH, SpCas9-NRCH and SpCas9-NRTH, where H is A, C or T, and R is A or G). In addition, researchers can use catalytically dead Cas9 (dCas9), which cannot cut DNA, but does bind to specific DNA sequences to up- or down-regulate gene expression. New CRISPR–Cas-derived genomic editing agents have also been developed, such as Base Editing and Primer Editing, which allow changes to be introduced into DNA without the need to produce a DSB. Base editing consists of a Cas9 nickase (nCas9-an enzyme that cleaves only one of the DNA strands) linked to a deaminase enzyme (cytosine deaminase or adenine deaminase) that allows nucleotide transition in the target sequence. Prime editing consists of an nCas9 linked to a reverse transcriptase enzyme that allows nucleotide transition and transversion or insertion and deletion of small DNA fragments in the target sequence (reviewed in [85]).

#### 1.2.4. Comparison of the Three Types of Genome Editing Technologies

Current gene editing techniques differ from one another in several ways such as target site recognition sequence, difficulty of delivery, nuclease size or off-target rate and have their respective pros and cons (summarized in Table 2).

##### Sequence Selection and Assembly Evaluation

ZFNs offer great versatility as they can be designed to target any sequence, nevertheless the specificity of individual zinc finger depends on two factor such as the context of the surrounding zinc fingers and DNA sequence [86]. Therefore, in practice, a suitable ZFN target sequence may not be found for a specific gene or chromosomal loci [69]. Furthermore, the assembly of ZFNs is complicated, involving laborious and time-consuming steps [74,92], and often assembled ZFNs fail to cleave target sites [86]. 

TALENs target selection is limited by the requirement that TALE binding sites should start with a thymidine residue (T), and it is sensitive to DNA methylation [87,93]. Not all TALENs are efficient to cleave target sites, and TALENs pairs must be experimentally validated [69]. The simple one-to-one specific recognition relationship between TALE repeats and the four nucleotides makes TALENs easier to design and assemble than ZFNs, but the assembly is still quite laborious and time-consuming [87]. One potential advantage over ZFNs is that the TALE repeat array can be easily extended to whatever length is desired [69]. Both ZFNs and TALENs rely on engineering proteins to specifically recognize target sequences and the complicated assembly process is the major hurdle preventing their wider application for genome editing [69].

In comparison, CRISPR-Cas9 target selection is limited by the presence of a PAM sequence downstream of the target sequence. Despite the PAM limitation, it is easy to find target sequences, for instance for the case of SpCas9 there is a PAM sequence (NGG) on average once every 8 bp. This simplicity is the main reason for the widespread adoption of the CRISPR-Cas9 systems and their application in numerous organisms [70].

##### Delivery Strategies

The major barrier for in vivo gene editing is the absence of safe and effective methods for local and systemic delivery of ZFNs, TALENs and CRISPR-Cas9 [94,95]. The delivery strategy must be able to specifically direct the genome editing complex to the target cell/tissue, and then this complex must be introduced into the cell, which requires crossing the plasma and nuclear membranes [96]. There are two strategies available to achieve this aim: viral and non-viral vectors (include physical and chemical methods) [95,96,97].

The most widely used method is viral vectors, in which nucleic acids coding for the protein complexes are packaged into viruses and delivered to the target cell. Nevertheless, viral vector cargo size is a limitation [96]. The typical size of a cDNA encoding a ZFN, a TALEN and a SpCas9 is approximately 1 kb, 3 kb and 4.2 kb in size, respectively. The CRISPR cDNA is somewhat larger than a TALEN monomer and much larger than a ZFN monomer (though both TALENs and ZFNs require dimerization, making their effective sizes larger) [69]. The size of the TALENs and Cas9 is a problem in the use of viral vectors that have a limited cargo size, such as adeno-associated virus (AAVs), with less than 5 kb [69,94,97]. However, to accommodate larger payloads, other viral vectors with larger capacities, such as adenoviral (AdVs) and lentiviral vectors (LVs), have also been investigated by various researchers for the gene-editing reagents delivery [88]. Nonetheless, these last two viral vectors have certain drawbacks such as immunogenicity and random integration, respectively [95], which would hinder their future application in gene therapy. Therefore, AAV is presented as the best option for its use in gene therapy in humans, as it does not develop immunogenicity.

Non-viral delivery methods have emerged as a viable alternative since they have the potential to solve the problem of cargo size and immunogenicity of viral vectors. However, the efficiency of delivery is slightly lower than that of viral vectors [98,99]. A major advantage of non-viral vectors is that they are artificially synthesized so that such important parameters as size, surface ligands, thermal properties, loading capacity, encapsulation efficiency and storage stability can be con-trolled [100,101]. What makes non-viral vectors even more promising is the rapid development of materials science applied to molecular biology, which will substantially facilitate the improvement of the efficiency and effectiveness of these non-viral vectors [99]. On the one hand, physical non-viral methods include microinjection, which involves injecting the genome editing complex directly into cells with a microscope and a needle; and electroporation, which applies pulses of electric current to stimulate the transient opening of pores in cell membranes, allowing delivery of the genome editing complex into cells [95]. On the other hand, non-viral chemical methods include lipid-based nanoparticles (LNPs), which involve encapsulation of negatively charged nucleic acids in positively charged liposomes for introduction into cells [102]; and cell penetrating peptides (CPPs), which are short peptides with an intrinsic ability to trans-locate across cell membranes, which is exploited to facilitate the delivery of a variety of cargoes into cells [103].

##### Specificity and Efficiency

An ideal gene editor would exhibit perfect specificity for the target sequence and cause no mutations to any other region of the genome. Unfortunately, ZFNs, TALENs and CRISPR-Cas9 rarely achieve such a high level of specificity and can generate mutations at the non-target locus (off-target activity) [104].

The specificity of ZFNs is determined by three major factors: the amino acid sequence of each ZF, the number of ZFs, and the interaction of the nuclease domain [89]. The specificity of TALENs is determined by RVDs, constituting a strikingly simple TALE–DNA recognition cipher [105,106]. Both ZFNs and TALENs tolerate a small number of positional mismatches, but TALENs exhibit better specificity than ZFNs [89]. The specificity of CRISPR-Cas9 is determined by the sgRNA (structure and composition) and PAM sequences [107,108,109]. CRISPR-Cas9 tolerates multiple consecutive mismatches and, compared to ZFNs and TALENs, CRISPR-Cas9 can exhibit higher off-target activity [89,110].

In addition to off-target activity, on-target effects must also be considered. This refers to large deletions, rearrangements or translations in the target sequence resulting from DSB repair induced by gene editing tools [111]. On-target effects are not dependent on the activity of nucleases but are associated with the functioning of DNA repair mechanisms in cells. To date, on-target effects cannot be predicted and may even be considered a bigger problem than off-target activity [112]. Therefore, on-target effects and off-target activity must be carefully monitored to avoid obtaining unintended gene modifications.

Efficiency is a difficult parameter to compare since, in many cases, it depends on the selected objective. In general, CRISPR-Cas9 (81%) has higher efficiency than ZFNs and TALENs, whereas TALENs (76%) usually exhibit higher efficiency than ZFNs (12%) [69,90].

## 2. Site-Specific Nuclease Systems to Study Genetic Kidney Diseases

**Figure 4:**
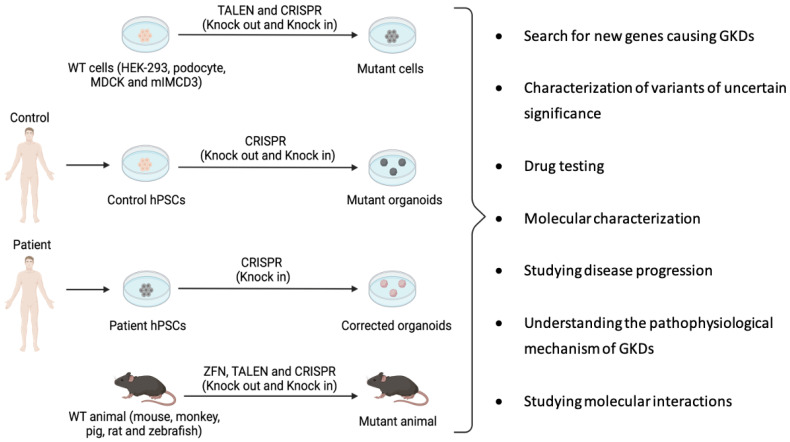
Workflow for disease modeling using genome editing technologies in different models. The different gene editing tools used in each of the possible models (cells, organoids, or animals) are represented, indicating their implication in the study of GKDs.

As previously mentioned, there is a profound lack of understanding of the pathophysiology and treatment of GKDs. The key to solving these problems is finding a suitable model (in vitro or in vivo) of these diseases for use in preclinical studies. Engineered nucleases (ZFNs, TALENs and CRISPR-Cas9) allow genome editing, which can be used to develop potentially useful models to study the progression and possible treatment of these human diseases. In addition, these nucleases enable find new genes involved in GKDs, since many of these genes remain unknown (Figure 4). The general approach is to knock out the gene responsible for the disease and to inspect the generated mutant models for phenotypic differences with isogenic controls (i.e., having a uniform genetic background) that were not modified by the nucleases [113].

There are numerous in vitro models to unravel the mechanisms of GKDs and perform drug screening tests with the aim of identifying a potential therapy for GKDs. In vitro models are available ranging from simple monolayer cultures of various renal cell lines to three-dimensional (3D) culture, and finally kidney organoids. The most common renal cell lines are Madin–Darby canine kidney (MDCK), inner medullary collecting duct (IMCD3) and human embryonic kidney cells (HEK293). In addition, human pluripotent stem cells (hPSCs) enable the production of organoids modeling human disease. Although these models clearly represent a very useful tool for understanding GKDs, it should be noted that due to the absence of their natural environment, these models are not representative of the physiology of the entire organism. Therefore, when interpreting the results of in vitro tests, it must be considered that these cell models lack important aspects, such as complex drug metabolization or tissue interactions. Hence, in vivo validation of results remains a mandatory step to understand the pathophysiological mechanisms of GKDs and drug development [114].

Just like in vitro models, a large number of in vivo models are available for the study of GKDs [115]. These models should recapitulate the phenotype observed in human pathology following the introduction of mutations in the orthologous gene corresponding to each type of GKDs [114]. In vivo models include invertebrate or lower vertebrate organisms, such as *Xenopus* or *Danio rerio*, useful for drug screening due to their fast generation time [116,117], as well as higher vertebrate organisms, especially mammals, which share greater genetic and physiological similarity with humans. Studies in the latter models better reflect human disease mechanisms and allow for more reliable observations, especially to determine treatment efficacy and adverse effects [114].

Although the field of GKDs is largely unknown, precision gene editing has been used in kidney research to achieve both gene knock-out and gene knock-in in a variety of model, from individual cells to animals complete (Figure 4; Table 3). These studies have generated new insights into the mechanisms of GKDs and have illustrated the potential of genome editing as a tool to enable progress in targeted gene therapy [118]. Below, gene editing tests carried out in different models for the study of GKDs are shown.

### 2.1. ADPKD Models Generated Using Genome Editing

ADPKD is the most common GKD and the most studied. There are several publications related to the application of genome editing for the study of ADPKD, most of them using CRISPR-Cas9 technology in organoids. The strategy was to knock-out *PKD1* or *PKD2* genes introducing InDels by NHEJ. However, studies have also been carried out using ZFNs and TALENs technologies in cells and animal models.

#### 2.1.1. ADPKD Models Generated Using ZFNs

In 2015, Jin He et al., created a mini pig model by mono-allelic knockout of *PKD1* gene by using ZFNs [119]. This model by 6 months of age macroscopically visible renal cysts appeared. These cysts grew in number and size and at 24 months these began to deform the normal kidney shape. Therefore, the *PKD1* mono-allelic knock out is sufficient to trigger renal cystogenesis. No piglets *PKD1*^−^/^−^ were born, perhaps since it resulted in embryonic lethality as occurs in human [143] and in some mouse models [144]. In addition, this model has been used for drug testing. In 2019, Lian et al., researched the effect of single and combined treatments with 2Deoxy-D-glucose and metformin on ADPKD progression. They conclude that both drug treatments significantly inhibited cystogenesis in the ADPKD mini pig model, being the combined therapy the most effective [145].

#### 2.1.2. ADPKD Models Generated Using TALENs

TALENs technology has been applied to study the role of *PDE1A* gene in the pathogenesis of ADPKD. *PDE1* encodes phosphodiesterase 1 (PDE-1), the only PDE activated by calcium and the main enzyme degrading cAMP in the distal nephron and CD. Substantial evidence supports the hypothesis that disruption of polycystin function results in dysregulation of intracellular calcium dynamics, producing the inhibition of PDE-1 and causing an increase in intracellular cAMP, thus contributing to the development and the progression of ADPKD [146,147,148]. To validate this hypothesis, Hong Ye and colleagues in 2016 and Xiaofang Wang et al., in 2017, developed a *Pde1a* knockout (KO) mouse model using TALEN tool [128,129]. They conclude that *Pde1a*^−^/^−^ aggravates cystic formation on a *Pkd2* mutant background. These results support an important role of *PDE1A* in the renal pathogenesis of ADPKD. In addition, this model may contribute to a better understanding of the mechanisms responsible for increased cAMP signaling in ADPKD and to the search for treatments capable of increasing PDE1 expression to delay cyst growth [128,129].

Furthermore, it is noteworthy the easily implementable workflow developed by Alexis Hofherr and coworkers in 2017 [120]. They applied TALEN and CRISPR systems for the rapid generation of targeted heterozygous and homozygous genomic sequence alterations in differentiated renal epithelial cells (MDCK and mIMCD3). In order to demonstrate the versatility of their genome editing approach, they established multiple novel cell lines for the study of ADPKD by introducing targeted mutations into *Pkd1* and *Pkd2*, generating deletions of *Pkd* genes, rescuing polycystin expression, and developing cell lines incorporating multiple allelic features [120].

#### 2.1.3. ADPKD Models Generated Using CRISPR-Cas9

CRISPR has been the most widely used tool to study ADPKD due to its cost-effectiveness. This tool has been mainly applied in organoids, with the aim of disease modeling and drug screening. Freedman and colleagues, in 2015, were the first to establish not only a protocol for differentiate hPSCs into kidney organoids (multicellular units containing podocytes, proximal tubules and distal tubules cells), but also the application of CRISPR in organoids to model GKDs [121]. They introduced frameshifts mutations by CRISPR-Cas9 in either *PKD1* and *PKD2* genes and established the first genetically edited models of disease in human kidney organoids. It is highly relevant that shortly after differentiation, organoids with *PKD* mutations formed cysts in vitro while no cysts were detected in control organoids with identical genetic backgrounds. In addition, using this protocol, the problem of variability among hPSCs from different patients to differentiate into kidney organoids disappears, as CRISPR generates series of *PKD1*/*PKD2* mutant hPSCs that are otherwise isogenic. Nevertheless, one limitation of this study was that only 6% of CRISPR-*PKD* organoids developed cysts [121].

Two years later, Cruz et al., improved cystogenesis (~75% of CRISPR-*PKD* organoids developed cysts) by growing CRISPR-*PKD* kidney organoids in suspension culture [122]. Taking advantage of this high percentage of cyst formation, kidney organoids could be a perfect tool for understanding the pathogenesis of ADPKD and for drug screening.

In 2020, Shohei Kuraoka and colleagues generated CRISPR kidney organoids and demonstrated that cAMP signaling is required for in vitro cystogenesis in ureteric bud (UB) organoids [123]. On the one hand, using CRISPR-Cas9 technology, *PKD1* gene was deleted in hPSCs and then *PKD1^+^/^−^* and *PKD1^−^/^−^* clones were induced to differentiate into UB organoids. On the other hand, they generated UB organoids from hPSCs from patients with ADPKD who had a heterozygous missense mutation. Subsequently, both types of UB organoids were treated with forskolin (an activator of cAMP signaling). CRISPR-*PKD1*^+^/^−^ and CRISPR-*PKD1*^−^/^−^ organoids exhibited cystogenesis upon forskolin treatment. In contrast, no cysts were formed in *PKD1*^+^/^+^ organoids after treatment. Similarly, *PKD1*^+^/^−^ organoids from patients exhibit cystogenesis upon treatment, but no cysts were formed in *PKD1*^+^/^+^ organoids. Although cAMP stimulation by forskolin may not accurately mimic cyst formation signals in vivo, UB organoids could be used to study the balance between cAMP and PC1/PC2 mediated signals that cause cyst formation [123].

Recently, Yasaman Shamshirgaran and coworkers established a faster protocol for generating genetically engineered kidney organoids [124]. To this end, they set out two modifications: (1) using a doxycycline-inducible Cas9 expressing hPSC line instead of transfecting them with Cas9-plasmid; and (2) using KO pools instead of generating isogenic clonal lines. Applying these procedures, they generated *PKD1* and *PKD2* mutant hPSC, both of which exhibited an editing frequency of 80%. Then, both pools of hPSCs were differentiated into kidney organoids. They observed cyst formation in approximately 50% of the *PKD* KO pools organoids, while a practically negligible rate of cysts was observed in control organoids (less than 1%). Therefore, by using KO pools instead of generating isogenic clonal lines, it is possible to generate cystogenesis in kidney organoids, providing a platform for rapid target validation in the context of disease modeling [124].

As shown, applying both hPSCs differentiation and CRISPR tool, is opening new opportunities for the study the genetic basis of ADPKD and the evaluation of new therapeutic options. For instance, in 2018, Czerniecki and colleagues treated kidney organoids derived from CRISPR-*PKD* hPSCs with blebbistatin, a specific inhibitor of non-muscle myosin II [149]. This treatment induced a significant dose-dependent increase in cyst formation, which revealed an unexpected role for myosin in ADPKD. The result suggests that the myosin pathway and the regulation of actin-myosin activation may be implicate in cystogenesis [149].

CRISPR technology can also be used to discover potential new disease-causing genes. Mutations in *PKD1* or *PKD2* were the known causes of ADPKD, but there was a percentage of families affected by ADPKD that were genetically unresolved (GUR). In 2016, Binu Porath et al., carried out genetic studies in GUR ADPKD-affected families and suggested that mutations in *GANAβ* gene (encodes glucosidase II subunit α -GIIα) produce ADPKD [14]. To validate this hypothesis, they knocked out *GANAβ* gene in renal cortical tubular epithelial (RCTE) cells by CRISPR-Cas9. The results showed that *GANAβ*^−^/^−^ cells exhibited failed trafficking of PC1 and PC2 to primary cilia. However, a normal localization of PC1 was rescued by wild type (WT) GIIα, but not by the mutant. In *GANAβ*^+^/^−^ a reduced mature PC1 was seen. Therefore, mutant GIIα cause maturation and localization defects of PC1 and PC2, and this may be related to cystogenesis [14].

Although kidney organoids are a very promising tool for the study of diseases, to date they have some limitations. Organoids lack vasculature so their growth is limited, they lack immune cells so they cannot be used to study processes that require inflammatory responses. Cellular composition varies significantly depending on the cell line and protocol used to generate organoids. Furthermore, when renal organoids self-assemble, tissues develop somewhat randomly, and hPSC-derived organoids show high variability. As existing culture methods cannot faithfully replicate renal conditions in vivo, the use of animal models is still necessary [150,151].

In 2019, Tomoyuki Tsukiyama and colleagues created cynomolgus monkeys with mutations in *PKD1* gene by CRISPR-Cas9 [125]. The resulting *PKD1*^+^/^−^ monkeys exhibited perinatal cyst formation in the distal tubules, possibly reflecting the initial pathology in humans. Many monkeys survived after cyst formation, and cysts progressed with age. However, no monkeys *PKD1*^−^/^−^ were born, which is consistent with the embryonic lethality observed in *Pkd1*-deficient mice, humans, and mini pigs [119,143,144]. Therefore, this model could elucidate the onset and progression of ADPKD, helping to establish new therapeutic strategies and discover new pharmacological treatments [125].

Masahito Watanabe et al., in 2022 applied CRISPR-Cas9 to generate heterozygous *PKD1* mutant pigs [126]. *PKD1*^+^/^−^ pigs had many symptomatic similarities to patients with ADPKD caused by heterozygous mutation of *PKD1* gene. For instance, presence of macroscopic renal cysts during the neonatal stage, cystogenesis process, interstitial fibrosis, and asymptomatic period during the first half of life. Taking advantage of these similarities between both, *PKD1*^+^/^−^ pigs can be used to study origin and development of this disease, check the effects of different treatment and test long-term treatments that cannot be carried out in rodent models [126].

It is important to note that although studies with cellular and animal ADPKD models have shed some light on this disease and have allowed potential treatments to be tested, such as Anti-TWEAK, which significantly slows disease progression, preserves renal function, and improves survival in an ADPKD mouse model. However, no definitive therapies are available [152]. Tolvaptan is the drug currently used in patients with ADPKD, it can slow the progression of renal cyst formation, but it is not capable of making them remit [153]. Therefore, more research of this disease is needed, and gene-edited cellular and animal models are essential.

### 2.2. ARPKD Models Generated Using Genome Editing

ARPKD is a less studied disease than ADPKD. Although, both are polycystic kidney disease and share their main renal symptom (cyst formation), ARPKD is much less prevalent than ADPKD due to its recessive nature. For the study of ARPKD, CRISPR technology has been the only gene editing tool that has been used.

#### ARPKD Models Generated Using CRISPR-Cas9

In 2018, Phillip Chumley and coworkers applied CRISPR technology in HEK-293 cell lines to generate diverse truncating mutations along multiple sites of *PKHD1* gene [127]. Their objective was to determine whether polyductin deficiency produces alterations in energy metabolism and proliferation rate, as occurs with polycystin 1 deficiency [154]. The results indicated that the *PKHD1*^−^/^−^ clones did not show a significant proliferation rate compared to the WT clones, but an increase in metabolism was observed. In parallel, they performed the same procedures for *PKD2* gene, but did not observe significant changes in the metabolism of *PKD2*^−^/^−^ clones. These results indicate that it is possible that the energy metabolism is altered in ARPKD, so this field could be studied to search for possible metabolism-related treatments [127].

The main application of CRISPR-Cas9 technology is to eliminate the expression of a certain gene through the formation of InDels. However, CRISPR can also be used to correct a specific mutation by introducing a donor oligo (WT sequence). In 2019, Low and colleagues applied the CRISPR-Cas9 gene editing tool in hPSCs from ARPKD patients to correct a specific mutation in *PKHD1* gene [130]. After forskolin treatment, ARPKD kidney organoids exhibited drastic cystogenesis. In contrast, CRISPR-corrected ARPKD kidney organoids showed only marginal cyst formation, as did WT hPSCs-derived kidney organoids [130]. Although the application of CRISPR technology as a mutation correction tool requires further study, this publication demonstrates the great potential of CRISPR-knockin and its possible application as gene therapy.

Renal defects in ARPKD heterozygous patients are very mild and poorly understood since renal tissue is not available as no biopsies are performed in these patients. To extend the knowledge about the impact of heterozygous mutations in this autosomal recessive disorder, in 2019, Dan Shan and coworkers generated heterozygous mutant *Pkhd1* mice by CRISPR [131]. Aged *Pkhd1*^+^/^−^ females mice developed ectasia of PTs, but not of DCTs, or CDs. These results suggest that patients with heterozygous mutations in *PKHD1* have a similar phenotype and develop only PT ectasia [131]. 

As occurs in ADPKD with *GANAβ* gene, in ARPKD there is also another gene that in a very small percentage can cause this disease. Mutations in *PKHD1* gene are the known causes of ARPKD, but in 2016, Hao Lu and coworkers identified patients with homozygous mutations in *DZIP1L* gene, who had a phenotype similar to patients with homozygous mutations in *PKHD1* (e.g., multiple small cysts) [28]. *DZIP1L* encodes cilium assembly protein DZIP1L, which through its interaction with septin2, participates in the maintenance of the periciliary diffusion barrier at the ciliary transition zone, allowing proper translocation of PC1/PC2. To validate that *DZIP1L* is a gene involved in the pathogenesis of ARPKD, they used CRISPR-Cas9 to develop a *DZIP1L* loss-of-function zebrafish model. They observed that zebrafish deficient for dzip1l had fewer motile cilia in the pronephric tubules. These results indicate that DZIP1L plays an important role in primary ciliogenesis and help to better understand the genetic heterogeneity underlying the pathogenesis of this disorder [28].

CRISPR-Cas9 technology can be applied to study the role of a specific gene in disease pathogenicity. For instance, the ionotropic P2X7 receptors are known to activate pannexin-1, a plasma channel capable of releasing ATP into the lumen. These receptors are thought to play a major role in cyst formation due to their high prevalence in the cyst wall (compared to healthy tissues) and the high ATP accumulation observed in cyst fluid [132,155]. To study mechanisms of P2X7 involvement in cystogenesis, Sergey N. Arkhipov et al., in 2019 generated a global KO of the *P2rx7* gene in PCK rats (a model of ARPKD) by CRISPR. They concluded that PCK-*P2rx7*^−^/^−^ rats showed slower cyst growth (but not slower formation of new cysts) compared to PCK-*P2rx7*^+^/^−^ and PCK-*P2rx7*^+^/^+^ rats [132]. These results support the involvement of ATP in cystic growth and highlight the role of P2X7 in the renal pathogenesis of ARPKD.

### 2.3. AS Models Generated Using Genome Editing

AS is the second most common genetic kidney disease and the second in which there are most publications related to genome editing. Nevertheless, as with ARPKD, CRISPR has been the only technology used to study this disease. The most widely used cellular model has been podocytes, which are the only cells that synthesize and secrete α3, α4 and α5 heterodimers in the glomerulus. These heterodimers are essential for GBM maintenance and mutations in the genes (*COL4A3*, *COL4A4* and *COL4A5*) encoding these proteins generate AS [35,156].

#### AS Models Generated Using CRISPR-Cas9

During the progression of AS, the number of podocytes is substantially reduced due to apoptotic processes. However, the mutational mechanisms that trigger this podocyte death are unknown. To shed light on this field, in 2019, Hui-Di Zhang and colleagues generated *Col4a3* KO mouse monoclonal podocytes using CRISPR [133]. They observed variations in the levels of endoplasmic reticulum (ER) stress-related proteins (increased expression of PERK, GRP94, CHOP, and phosphor-eIF2a) and in the levels of apoptosis-related proteins (increased expression of cleaved caspase 3 and decreased expression of Bcl-2) [133]. These results suggest that podocyte death could be triggered by dysregulation of both pathways due to *COL4A3* deficiency.

The following year, Sergio Daga et al., corrected a specific mutation in *COL4A3* and *COL4A5* genes in podocyte from two AS patients by Cas9 and an oligo donor [134]. They used an innovative self-inactivating dual-plasmid system, one plasmid carried the donor DNA and the variant-specific sgRNA, while the other plasmid carried a self-cleaving SpCas9. Applying these procedures, they generated CRISPR-corrected *COL4A3* and *COL4A5* podocytes, which showed a homology-directed repair frequency close to 50%. Although podocytes are not renewable, these results demonstrate the ability of CRISPR to correct a mutation and it is likely that the application of this technology in the early stages of the disease, when podocytes are still present, would be effective in treating this disease [134].

Most of the mutations described in *COL4A5* gene are missense mutation and most are described as likely pathogenic with uncertain significance [135,157]. To find out if the variant is responsible for the disease, CRISPR technology can be used to knock in the variant into the gene of interest and study how it changes the phenotype of the model. In 2021, Lei Sun et al., followed this strategy and introduced a missense mutation of the *COL4A5* gene (observed in a patient) into immortalized human podocyte cell lines to confirm whether this variant could cause abnormal collagen alpha-5 (IV) chain expression and consequently cause AS [135]. They concluded that this variant significantly reduced α5 chain expression in podocytes. These results highlight the possibility of using CRISPR-knockin as a tool to confirm the pathogenicity of missense variants in vitro [135].

Regarding the use of genome engineering in mouse models, CRISPR technology has been used to develop a murine model harboring a nonsense mutation (observed in one patient) in the *Col4a5* gene (*Col4a5*-R471X). In 2019, Kentarou Hashikam and coworkers generated male *Col4a5* hemizygous mutant mice, which exhibited pathologies similar to those observed in AS patients [136]. Among these pathologies were increased levels of urinary albumin and blood urea nitrogen (BUN). These findings are indicative of impaired renal function. Since the mechanisms of progression are unknown and there is no effective treatment for AS, this animal model could be useful for studying the mechanisms involved in the development of AS and for preclinical trials to restore adequate renal function [136]. 

As in other diseases such as ADPKD with *PDEA1*, in AS there are also other genes that by themselves do not cause the disease, but the presence of mutations in these genes in patients with mutations in *COL4* genes, aggravates the pathogenicity of the disease. An example would be the *LAMB2* gene encoding for Laminin b2, which is a major component of the GBM. In 2018, Steven D. Funk and collogues generated mice with a specific mutation in *Lamb2* gene (*Lamb2*-S80R) by CRISPR-knockin technology [137]. The results showed that the *Lamb2*^S83R/S83R^ and *Lamb2*^S83R/−^ mice did not exhibit a pathogenic phenotype. However, *Lamb2*^S83R/+^ significantly increased the rate of progression to kidney failure on a *Col4a3*^−^/^−^ background and increased proteinuria in females with a *Col4a5*^+^/^−^ background. These data demonstrate how the phenotype associated with AS can vary due to mutations in other genes that also encode GBM components. Furthermore, this mouse model could help explain the wide range of phenotypes observed in AS patients, even in those who share the same *COL4* mutation [137].

### 2.4. ADTKD Models Generated Using Genome Editing

ADTKD is the third most common monogenic kidney disease after exclusion of ADPKD and AS. This disease can be caused by mutations in five different genes; however, gene editing technologies have not been applied to all of these genes. ZFN and CRISPR have been the tools used to study this disease.

#### 2.4.1. ADTKD Models Generated Using ZFNs

*REN* gene, which encodes for renin, is one of the causative genes for ADTKD. Renin is known to play an important role in renal function. To shed light on the importance of renin in the context of ADTKD in vivo, Carol Moreno et al., in 2018 used ZFN technology to knock out *Ren* gene in SS rats (rodent model of Salt-Sensitive hypertension) [138]. They observed that *Ren*^−^/^−^ SS rats had elevated BUN levels (indicative of poor renal function), abnormal renal morphology with cortical interstitial fibrosis and incorrect orientation of CDs and DCTs. These results highlight the importance of renin for proper renal morphology and function [138]. 

#### 2.4.2. ADTKD Models Generated Using CRISPR-Cas9

As previously shown with other genes such as *PKD1* and *PKD2*, the combination of kidney organoids and CRISPR technology is ideal for studying how deletion of a given gene influences renal development and disease [121,122,124]. In this sense, Aneta Przepiorski and coworkers in 2018 knocked out the *HNF1β* transcription factor gene in renal organoids by CRISPR-Cas9, which is known to be one of the genes responsible for ADTKD and plays an essential role in tubulogenesis [139]. *HNF1β*^−^/^−^ organoids showed reduced levels of markers associated with PT (LRP2) and TAL (UMOD, SLC12A1). These results indicate that KO of *HNF1β* prevents proper formation of the PT and TAL segments [139]. This is consistent with that described in the *Hnf1b* KO mouse [158]. However, cyst formation was not observed in *HNF1β*^−^/^−^ kidney organoids, even after several weeks in culture [159]. This indicates that, although organoids are useful for studying human kidney development, more research is needed for organoids to more accurately mimic the ADTKD phenotype.

As already mentioned, CRISPR technology can be used to discover new disease-causing genes. Until 2016, the known ADTKD-causing genes were *REN*, *UMOD*, and *MUC1*. In that year, Nikhita Ajit Bolar and colleagues identified two families with heterozygous missense variants in *SEC61A1* gene and attempted to determine the pathogenicity of the missense variants in vivo [55]. To do so, they applied CRISPR technology to delete the human *SEC61A1* ortholog, *sec61al2*, in zebrafish. Zebrafish showed defects of the pronephric tubules (consistent with the tubular atrophy observed in affected individuals), confirming that the SEC61 complex and its translocation function are required for normal renal development. These results highlight the role of *SEC61A1* in the formation of the nephron and broaden the spectrum of ADTKD-causing genes [55].

*UMOD* gene, which encodes for uromodulin, is another ADTKD-causing gene. However, its role in disease progression is unknown. Studies suggest that it protects against uropathogenic bacteria, controls water transport in the kidney to concentrate urine [159,160] and is critical in preventing crystallization of calcium from the tubular filtrate [161]. In patients, mutant uromodulin has been shown to be poorly secreted and to accumulate in the ER of the distal renal epithelium. To further explore the signaling pathways triggered by misfolded uromodulin, Bryce G. Johnson et al., in 2017, developed a mouse model carrying a mutation in *Umod* gene (observed in patients) using CRISPR and an oligo donor [140]. *Umod*^+^/^−^ mice exhibited renal failure at 24 weeks (elevated BUN and serum creatine levels), decreased body weight, renal fibrosis, reduced urinary uromodulin levels and accumulation of uromodulin in the ER, which activates unfolded protein response mechanisms and triggers ER stress-dependent cell death in renal tubule epithelial cells. This murine model recapitulates many features of ADTKD-*UMOD* patients and provides critical insight into signaling pathways altered in the pathogenesis of this disease. This makes it a valuable animal model for future studies [140].

### 2.5. GS and BS Models Generated Using Genome Editing

GS and BS are the least prevalent of the diseases discussed in this review. Moreover, they are those in which there are fewer publications related to the application of gene editing tools. On the one hand, no gene editing studies have been performed in GS. On the other hand, although BS is less prevalent than GS, two gene editing studies have been performed, one with ZFNs and the other with TALENs. 

#### 2.5.1. BS Models Generated Using ZFNs

*KCNJ1* gene, which encodes for ATP-sensitive inward rectifier potassium channel 1, is one of the causative genes for BS. It mediates potassium recycling and facilitates sodium reabsorption in the TAL and potassium secretion in the cortical CD [162]. To investigate the effects of knocking out *KCNJ1* gene on systemic and renal hemodynamics, in 2013, Xiaoyan Zhou and coworkers developed *Kcnj1*^−^/^−^ and *Kcnj1*^+^/^−^ SS rats by ZFNs [141]. SS rats *Kcnj1*^−^/^−^ pups recapitulated many of the features of BS in humans such as severe volume depletion, increased BUN, hyperkalemia, and metabolic acidosis. *Kcnj1*^+^/^−^ and *Kcnj11*^+^/^+^ SS rats exhibited the same phenotype when fed a low-salt diet, but when fed a higher-salt diet, *Kcnj1*^+^/^−^ SS rats exhibited a reduced blood pressure (BP) and signs of protection from renal injury, compared with the *Kcnj1*^+^/^+^ littermates. These results are also observed in humans with *KCNJ1* heterozygous mutation, but the mechanism of BP reduction by *KCNJ1* disruption is not entirely understood. Nevertheless, since *KCNJ1* plays a critical role in the TAL and in the cortical CD, it has been hypothesized that kidney has a central role in BP control. Future studies using this rat model would help to further understand the role of *KCNJ1* in hypertension and renal injury [141].

#### 2.5.2. BS Models Generated Using TALENs

Another gene that causes BS is *CLCNKB*, which codes for chloride channel protein ClC-Kb, and its function is to reabsorb chlorine in the TAL [60,163]. Chlorine reabsorption by ClC-Kb is thought to facilitate transepithelial salt reabsorption in the TAL [164]. Therefore, compromised ClC-Kb function could result in severe salt-losing nephropathy. To evaluate the reabsorptive and regulatory function of ClC-Kb in vivo, Alexandra Grill and colleagues, in 2016, used TALEN technology to generate *Clcnk2* KO mouse lines (orthologous gene of *CLCNKB* in mice) [142]. ClC-K2-deficient mice showed a phenotype similar to that of BS patients, such as marked diuresis and low urinary concentrating ability, accompanied by a reduced salt-retaining capacity. These data suggest that ClC-K2 has an important role in TAL transepithelial salt transport and renal concentrating ability [142].

## 3. Main Challenges to Overcome

All this research demonstrates how extremely useful the application of site-specific nucleases systems in cellular and animal models can be in gaining more information about GKDs. Despite the promise, there are multiple obstacles to accurate an efficient in vivo genome editing in kidney. These challenges include: the ability of the vectors to effectively introduce the gene editing machinery into renal cells, and the immune response against the newly expressed proteins, and against the vector itself.

Evidence of the difficulty of performing renal gene editing in vivo is that in all of the publications cited above using animal models, gene editing was performed ex vivo and not in vivo. The strategies used were zygote injection (i.e., injection of the construct of interest into the pronucleus of fertilized eggs, and then these injected eggs are implanted into the oviduct of a pseudopregnant adoptive mother) or somatic cell nuclear transfer (i.e., transfer of the nucleus of a somatic cell into the cytoplasm of an enucleated egg, becoming the nucleus of the zygote, and then this zygote is implanted into the oviduct of a pseudopregnant adoptive mother).

Overcoming these challenges would not only facilitate the development of in vivo models of GKDs but would also open the door to the possible use of these technologies as a potential gene therapy against GKDs. There is evidence that the kidney has the capacity for plasticity. In 2021, Ke Dong et al., demonstrated that re-expression of *Pkd1* or *Pkd2* genes results in rapid reversal of ADPKD, as cystic cell proliferation and inflammation are reduced, kidneys regain their normal size, and renal function improves [165]. These promising results suggest that ADPKD, as well as other GKDs in which renal integrity and function are compromised, could be treated with gene therapy to correct the disease-causing mutation.

### 3.1. Difficulty of Gene Delivery to the Kidney

There are two main problems for efficient delivery of the gene editing machinery by vectors to renal cells: the access problem and the tropism problem. The access problem refers to whether the vectors can reach the target cells. The tropism problem considers whether, if the access problem is overcome, the vectors can interact with the target cells in the kidney [166].

The access problem is due, in part, to the stringent filtering functions intrinsic to the kidney. This filtration takes place in the glomerulus (specifically in the glomerular filtration membrane-GFM) of each nephron [166]. Blood, via the afferent arteriole, reaches the glomerulus, where molecules with a weight greater than 50 kDa and a size greater than 10 nm are excluded [166,167,168]. Blood is drained from the glomerulus through the efferent arteriole that branches forming the peritubular capillaries (PTCs), which extend along the PT and DCT, producing the exchange of substances. Finally, the PTCs flow into arcuate vein and the blood leaves the kidney through the renal vein [167].

**Figure 5:**
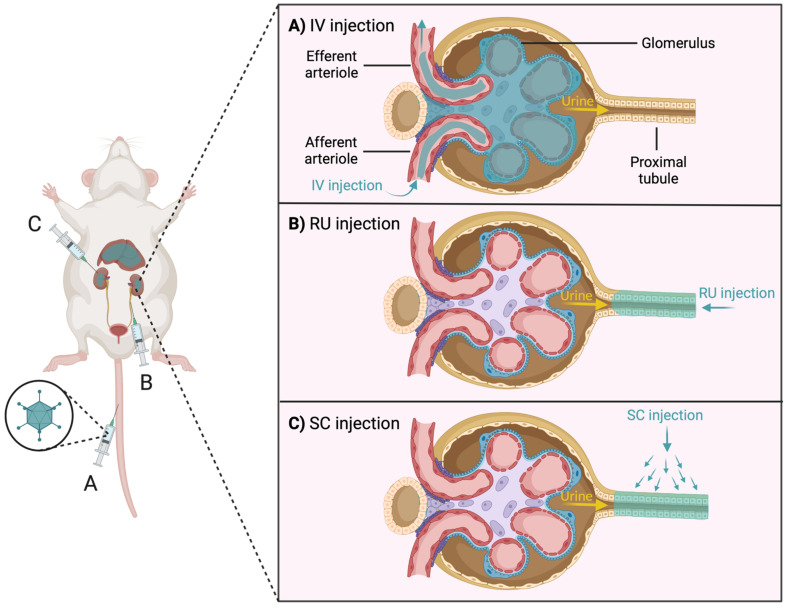
Expected target for infection in the context of the organism and the renal corpuscle after intravenous and direct kidney injection of typical viral vectors. Injection of viral vectors by any of the three strategies would infect renal cells, but mainly hepatocytes (left); (**A**) intravenous (IV) injection of viral vectors would cause only glomerular cells to be infected or recirculate into the bloodstream, due to the strict filtering functions of the glomerulus; (**B**) retrograde ureteral (RU) injection of viral vectors would avoid the filtering effects of the glomerulus but would be faced with going upstream against the natural flow of urine, although it would theoretically allow the vectors access to the tubules; (**C**) subcapsular (SC) injection of viral vectors would bypass the glomerulus and urinary flow and allow the vectors access to the tubules. Transduction is depicted with tissues and cells shaded in blue. Adenoviral vector (AdV).

Since most viral and non-viral vectors are larger than 10 nm in diameter and megadaltons in size, intravenous (IV) injection (Figure 5A) of vectors is not a useful strategy for the kidney, as they would not be able to cross the GFM [168]. It has been shown that after IV administration of AAV (the smallest viral vector, 25 nm), these were not able to cross the GFM and only glomerular cells were infected [166]. Therefore, IV injection of vectors seems to be relevant for the study of glomerulopathies, such as Alport syndrome, but not for other ERGs (e.g., tubulopathies) [166].

It is noteworthy that although these vectors cannot reach tubular cells by crossing the GFM, there is the possibility of reaching tubular cells through PTCs. It has been published that by injection of naked plasmid DNA into the renal vein, it is possible to transfer it to interstitial fibroblasts near PTCs, but not to tubular cells [169,170]. It has also been reported that by IV injection of nanoparticles (20 and 100 nm in size), these accumulated in the glomerulus and PTCs, but not in the tubules [171]. However, promising results show that after IV injection of mesoscale nanoparticles (~400 nm in size), they were internalized by the proximal tubule epithelial cells at basal side via passing through the peritubular capillaries [172]. Another study showed that, after IV injection of nanoparticles (140 nm iron oxide cubes and clusters, with PEG), they were able not only to penetrate the tubular cells but also to reach the lumen and be excreted via urine [173]. Nevertheless, further studies are needed to elucidate the mechanisms of translation and excretion of these nanoparticles that are larger than the GFM limit.

To avoid the GFM limitation there is the possibility of injecting the vectors directly into the kidney by two strategies: (1) retrograde ureteral (RU) injections (Figure 5B), avoiding the filtration effect of the glomerulus but with the limitation of going upstream against the natural flow of urine or (2) subcapsular (SC) injections (Figure 5C), both limitations are avoided, and the infection of the tubular cells is improved [166]. However, it has been observed that in each of the three injection strategies (IV, RU and SC), infection levels in other organs such as the liver were elevated, highlighting the problem of vector tropism [166,168,174,175].

The tropism problem greatly conditions efficient delivery to the kidney. Most vectors used for in vivo gene delivery have the liver or spleen as their first target organ, making it very difficult for the vector to reach the kidney [118]. For instance, after IV injection, the liver absorbs approximately 98% of the injected AdVs [176,177]. To solve the problem of vector tropism, vector modification may be a strategy to pursue. By adding certain peptides to the viral capsid, their specificity for the kidney can be increased, diverting their attraction to the liver [166]. In addition, off-target expression of the transgene can be restricted using kidney-specific promoters, such as *Ksp-cadherin* gene promoter [178]. However, delivery of the gene editing machinery outside the primary target, the kidney, may not pose substantial safety concerns. It would even be beneficial to some diseases such as ADPKD or ARPKD, in which the liver is also affected (liver cyst formation) [179].

In general, AdVs and AAVs are the most robust viral vectors for in vivo gene delivery. Several serotypes of these vectors exist, varying in target cell specificity and immunogenicity. The most used serotype of AdVs for renal research is AdV5, although this serotype is also largely taken up by the liver following IV injection [168]. Since adenoviruses are non-enveloped and can be retargeted by genetic or chemical modifications of their various capsid proteins [180,181], attempts have been made to increase the tropism of AdV5 for the kidney. For this purpose, peptides were introduced into the HI loop, which is a C-terminal region of the fiber protein (its function is to allow the adenovirus to bind to the target cell) [182,183,184]. Using this approach, it was possible to increase the infection of renal cells after IV injection [168]. 

In relation to AAVs, there is a wide variety of serotypes (e.g., AAV1, 2, 5, 6, 7, 8, 9). The serotypes with the best efficiencies for delivery in the kidney are 6 and 8 [166,185]. However, as with AdVs, a large leakage of AAVs to the liver is observed, even when injected directly into the kidney. A possible solution to this problem is the use of clamps (i.e., clamping of the renal artery, renal vein, and ureter to increase the time of physical exposure of AAVs in the injection fluid to kidney cells), which has markedly increased renal cell infection [166,174]. These results indicate that clamps may be a good way to increase and restrict infection to the kidney.

Although it has been shown here that there are different solutions to the problems posed for the delivery of genetic material into the kidney through the different types of existing vectors, there is still much room for improvement. Overcoming the delivery problem will certainly facilitate the development of more animal models to increase knowledge about GKDs.

### 3.2. Immune System against Gene Editing

As mentioned above, viral vectors are currently the most efficient and widely used platform for delivering gene editing machinery to target cells. In addition, many of the proteins used in gene editing are derived from bacteria (e.g., SpCas9). The fact that the components necessary to perform in vivo gene editing are foreign to the immune system may stimulate immune responses through three main pathways: innate immunity, humoral immunity, or cellular immunity [186]. This immune response further limits the efficacy of gene editing in renal cells.

Innate immunity uses pattern recognition receptors to recognize conserved features in microbes [187]. This raises the possibility that the viral vector may be recognized and eliminated [104]. Humoral immunity is mediated by antibodies, which can eliminate engineered nucleases or delivery vectors. It has been shown in studies in different animal models that administration by viral vectors of SpCas9 or SaCas9 results in the development of antibodies against these proteins [188,189,190]. Finally, cellular immunity is mediated by cytotoxic T cells, which can eliminate cells expressing the engineered nucleases. As with humoral immunity, studies in animal models have demonstrated the development of cellular immunity to Cas9 after delivery of SpCas9 and SaCas9 by viral vectors [188,189,190].

Therefore, for gene editing to be effective, it is important to determine and control the immune response against the delivery vector as well as against the engineered nucleases. A possible solution to reduce immunogenicity against these components is to identify the epitopes that cause immune stimulation, to modify them and thus reduce their immunogenicity [104]. Another possible solution is to replace the use of viral vectors with non-viral vectors. Noteworthy is the use of LNPs, which are a clinically approved drug delivery system [191]. LNPs present a matrix containing soluble lipophilic molecules, which gives them the advantage of interacting better with cells and thus facilitating greater cellular uptake of the transported drug [192]. Like non-viral vectors, it has the disadvantage that it accumulates naturally in the liver, so it is necessary to include modifications to ensure good uptake in other cell types [193]. Regarding the kidneys, the size and charge of LNPs considerably influences delivery efficiency (e.g., small LNPs penetrate more easily into the kidneys but are more easily eliminated) [194,195]. Although further research is needed to increase the efficiency and specificity of LNPs for the kidney, these systems have been successfully used in several studies for drug delivery to the kidney [196,197,198,199].

## 4. Conclusions

Gene editing systems based on site-specific nuclease are a versatile tool offering a multitude of applications for the study of genetic diseases. ZFNs, TALENs, and CRISPR-Cas9 have allowed the establishment of several in vitro and in vivo models that mimic the GKDs observed in patients, helping to improve the understanding of the pathophysiological mechanisms of GKDs, discover new disease-causing genes, resolve variants of uncertain significance, and drug testing. However, the application of these technologies as a possible treatment in the field of GKDs has not yet been successful due to the lack of knowledge about on-target and off-target effects and the difficulty of in vivo gene delivery into the kidney. The selection of an appropriate gene delivery method is crucial to optimize the efficacy of genome editing in the kidney. Currently, the most commonly used vectors are of viral origin and present problems of access and tropism to the kidney and may trigger an adverse immune response. It is worth trying to overcome these problems to facilitate and promote the successful establishment of more GKDs in vivo models that will contribute to generating new knowledge on disease development and progression. Furthermore, given the demonstrated plasticity capacity of the kidney, gene therapy based on gene editing tools could be a future option for treating Genetic kidney diseases.

## Figures and Tables

**Table 1:** List and classification of Genetic kidney diseases reviewed in the text.

Disease	Estimated Incidende (per 100,000 Population)	Gene	Protein	Function
Autosomal dominant polycystic kidney disease (CL)	~100 individuals	*PKD1* *PKD2* *GANAβ* *DNAJB11* *IFT140*	Polycystin-1 Polycystin-2 Glucosidase II subunit α DnaJ homolog subfamily B member 11 Intraflagellar transport protein 140 homolog	Calcium ion transmembrane transport Calcium ion transmembrane transport Alpha-glucosidase activity Misfolded protein binding Cilium assembly
Autosomal recessive polycystic kidney disease (CL)	~5 individuals	*PKHD1* *DZIP1L*	Polyductin Cilium assembly protein DZIP1L	Cell-cell adhesion Metal ion binding
Alport syndrome (GL)	~50 individuals	*COL4A3* *COL4A4* *COL4A5*	Collagen alpha-3 (IV) chain Collagen alpha-4 (IV) chain Collagen alpha-5 (IV) chain	Extracellular matrix organization
Autosomal dominant tubulointerstitial kidney disease (TL)	N/A	*UMOD* *MUC1* *REN* *HNF1B* *SEC61A1*	Uromodulin Mucin-1 Renin Hepatocyte nuclear factor 1β α1 subunit of the SEC61	Cellular defense response DNA damage response Cell maturation Transcription factor Endoplasmatic reticulum organization
Gitelman syndrome (TL)	~2 individuals	*SLC12A3*	Solute carrier family 12 member 3	Ion transport
Bartter syndrome (TL)	<1 individual	*SLC12A1* *KCNJ1* *CLCNKA* *CLCNKB* *BSND* *MAGED2*	Solute carrier family 12 member 1 ATP-sensitive inward rectifier potassium channel 1 Chloride channel protein ClC-Ka Chloride channel protein ClC-Kb Barttin Melanoma-associated antigen D2	Ion transport Potassium ion transport Chloride transport Chloride transport Chloride transport Sodium ion absorption

Abbreviations: CL: ciliopathy; GL: glomerulopathy; TL: tubulopathy [11]. N/A: not available.

**Table 2:** Comparison of ZFNs, TALENs and CRISPR-Cas9.

	ZFNs	TALENs	CRISPR-Cas9	References
Recognition site	Zinc finger motif	RVD region of tandem TALE repeat	Single-strand guide RNA	[72,77,80]
Endonuclease	FokI nuclease	FokI nuclease	Cas9 nuclease	[72,77,80]
Target sequence size	9–18 bp per ZFP monomer	14–20 bp per TALE monomer	20 bp guide sequence	[71,79,83]
Targeting limitations	Difficult to target non-G-rich sites	5ʹ targeted base must be a T for each TALEN monomer	Targeted site must precede a PAM sequence	[69,86,87]
Delivery (in vivo)	AAVs, LVs, AdVs	LVs, AdVs	AAVs, LVs	[69,88]
Specificity	Tolerating a small number of positional mismatches	Tolerating a small number of positional mismatches	Tolerating multiple consecutive mismatches	[89]
Efficiency	~12%	~76%	~81%	[69,90]
Cost (Single experiment)	$5000	$500	$30	[91]
Overall evaluation	Good	Very good	Excellent	

Abbreviations: ZFNs, zinc finger nucleases; TALENs, transcription activator-like effector nucleases; CRISPR-Cas9, clustered regularly interspaced short palindromic repeats-CRISPR associated protein-9 nuclease; AAVs, adeno-associated virus; LVs, lentiviral vectors; AdVs, adenoviral vectors.

**Table 3:** Use of site-specific nuclease systems in Genetic kidney diseases research.

Gene	Tool	Model	Key Finding	Refs.
**Autosomal dominant polycystic kidney disease**				
*PKD1*	ZFN	Mini pig	Heterozygous *PKD1* mini pigs develop cysts.	[119]
	TALEN and CRISPR	MDCK and mIMCD3	Protocol for creating knockout cell lines	[120]
	CRISPR	Kidney organoids	Knockout of *PKD1* causes cysts	[121]
	CRISPR	Kidney organoids	Growing kidney organoids in suspension culture enhances cystogenesis	[122]
	CRISPR	UB organoids	cAMP signaling is involved in cystogenesis	[123]
	CRISPR	Kidney organoids	By using knockout pools it is possible to generate cystogenesis	[124]
	CRISPR	Monkey	Heterozygous *PKD1* monkeys show cystogenesis perinatally	[125]
	CRISPR	Pig	Heterozygous *PKD1* pigs develop many pathological conditions similar to ADPKD patients	[126]
*PKD2*	TALEN	MDCK and mIMCD3	Protocol to creation knockout cell lines	[120]
	CRISPR	Kidney organoids	Knockout of *PKD2* causes cysts	[121]
	CRISPR	Kidney organoids	Growing organoids in suspension culture enhances cystogenesis	[122]
	CRISPR	Kidney organoids	By using knockout pools it is possible to generate cystogenesis	[124]
	CRISPR	HEK-293	Knockout of *PKD2* does not alter energy metabolism	[127]
*Pde1a*	TALEN	Mouse	Knockout of P*de1a* aggravates cystogenesis	[128]
	TALEN	Mouse	Knockout of P*de1a* aggravates cystogenesis	[129]
*GANAβ*	CRISPR	RCTE	Knockout of *GANAβ* causes PC1 and PC2 maturation and localization defects	[14]
**Autosomal recessive polycystic kidney disease**				
*PKHD1*	CRISPR	HEK-293	Knockout of *PKHD1* alters energy metabolism	[127]
	CRISPR	Kidney organoids	CRISPR-knockin as a method to correct pathogenic variants.	[130]
	CRISPR	Mouse	Heterozygous *Pkhd1* develop proximal tubule ectasia	[131]
*dzip1l*	CRISPR	Zebrafish	DZIP1L is involved in the formation of primary cilia	[28]
*P2rx7*	CRISPR	Mouse	P2X7 contributes to cyst growth by increasing pannexin-1-dependent ATP release into the lumen	[132]
**Alport Syndrome**				
*COL4A3*	CRISPR	Mouse podocytes	Knockout of *Col4a* causes endoplasmic reticulum stress and apoptosis	[133]
	CRISPR	Human podocytes	Innovative protocol for *COL4A3* correction by HDR	[134]
*COL4A5*	CRISPR	Human podocytes	Innovative protocol for *COL4A5* correction by HDR	[134]
	CRISPR	Human podocytes	CRISPR-knockin as a method for confirming the pathogenicity of missense variants	[135]
	CRISPR	Mouse	Heterozygous *Col4a5* male mice develop many pathological conditions similar to AS patients	[136]
*Lamb2*	CRISPR	Mouse	Heterozygous mutations in a gene encoding GBM components aggravate AS phenotype	[137]
**Autosomal dominant tubulointerstitial kidney disease**				
*Ren*	ZFN	SS rat	Knockout of *Ren* causes poor renal function	[138]
*HNF1B*	CRISPR	Kidney organoids	Knockout of *HNF1B* prevents proper formation of certain components of the nephron	[139]
*sec61al2*	CRISPR	Zebrafish	Mutations in *sec61al2* causes pronephric tubules defects	[55]
*Umod*	CRISPR	Mouse	Heterozygous *Umod* mice develop many pathological conditions similar to ADTKD-*UMOD* patient	[140]
**Gitelman and Bartter syndromes**				
*Kcnj1*	ZFN	SS rat	Knockout of *Kcnj1* protects against salt-induced hypertension and renal injury	[141]
*Clcnk2*	TALEN	Mouse	ClC-K2-deficient mice develop many pathological conditions similar to BS patient	[142]

Schematic representation of publications related to the application of gene editing tools in GKDs research, grouped by type of disease.
